# Effect of combined therapies including nutrition and physical exercise in advanced cancer patients: A pooled analysis

**DOI:** 10.3389/fnut.2023.1063279

**Published:** 2023-03-01

**Authors:** Lena J. Storck, Alexandra Uster, Lucia Gafner, Maya Ruehlin, Sabine Gaeumann, David Gisi, Martina Schmocker, Peter J. Meffert, Reinhard Imoberdorf, Miklos Pless, Peter E. Ballmer

**Affiliations:** ^1^Zentrum für Allgemeine Innere Medizin, Ernährungstherapie/-Beratung, Kantonsspital Winterthur, Winterthur, Switzerland; ^2^Medizinische Kliniken, Klinikum Konstanz, Konstanz, Germany; ^3^Division of Research, Innovation, and Development, Swiss Cancer League, Bern, Switzerland; ^4^Institut für Therapien und Rehabilitation, Kantonsspital Winterthur, Winterthur, Switzerland; ^5^Corvus, Statistical Analysis Consulting, Altkalen, Germany; ^6^Zentrum für Allgemeine Innere Medizin, Klinik für Innere Medizin, Kantonsspital Winterthur, Winterthur, Switzerland; ^7^Klinik für Medizinische Onkologie und Hämatologie, Kantonsspital Winterthur, Winterthur, Switzerland; ^8^Past President GESKES-SSNC, Winterthur, Switzerland

**Keywords:** cancer, cachexia, malnutrition, dietary counseling, physical exercise

## Abstract

**Background and aims:**

Although many cancer patients suffer from malnutrition or cancer cachexia, there is no standard of care so far due to limited intervention trials. Pooled data from two combined trials were analyzed regarding nutritional status and survival time.

**Materials and methods:**

Data from two trials with advanced cancer patients were included. In both trials, patients in the intervention group received at least three times nutritional counseling and supervised training sessions. Patients in the control group continued being treated according to usual care. Nutritional status was measured using BMI, body composition and handgrip strength. Survival time was analyzed using the Cox proportional hazard model with the period between the beginning of the trial and death as underlying time scale.

**Results:**

68 men (61.8%) and 42 women (38.2%) were randomized either to the intervention (*n* = 56) or the control (*n* = 54) group. The inter-group difference for changes in BMI and body composition was not statistically significant after 3 months. Handgrip strength improved significantly from 34.4 ± 10.2 kg to 36.3 ± 9.9 kg at 3 months in the intervention compared to 33.9 ± 9.2 kg to 34.9 ± 9.1 kg in the control group (*p* = 0.006). The analysis of survival time showed no inter-group difference for all patients. A detailed analysis for different diagnoses showed that in patients with lung cancer, the covariates “CRP value,” “days from first diagnosis to randomization” as well as “gender” were significantly associated with survival time. Patients with higher CRP value had a shorter survival time and female patients had a shorter survival time than male patients in our analysis. In addition, patients with pancreatic cancer randomized to the control group had a 20% shorter survival time than those in the intervention group (*p* = 0.048).

**Conclusion:**

The pooled analysis showed a significant improvement of handgrip strength in advanced cancer patients through the implementation of a combined therapy. Handgrip strength is of prognostic significance in hospitalized patients due to its association with mortality and morbidity. However, no improvements in further tests were detected. There is great need for further investigations examining the effect of nutritional and exercise therapy on survival time with focus on different cancer diagnoses.

## 1. Introduction

Approximately half of all tumor patients experience involuntarily weight loss during or even before their disease and suffer from malnutrition or cancer cachexia, especially patients with gastrointestinal cancer (1–3). Cancer cachexia is defined as “a multifactorial syndrome characterized by ongoing loss of skeletal muscle mass (with or without loss of fat mass) that cannot be fully reversed by conventional nutritional support and leads to progressive functional impairment” (4). Therefore, the consequences of malnutrition and cachexia are a substantial impact on quality of life (QoL), impaired functional status, reduced therapy tolerance, and an increased number of unplanned hospital admissions (5, 6).

Although many patients are affected by malnutrition or cachexia, there is no therapy and no standard of care so far (7). Since the pathophysiology of cachexia is complex, therapeutic approaches are intensely studied with a research focus on combined or multimodal therapy (8, 9). In recent years, research activity on this topic has increased significantly, which is also evident in numerous systematic reviews. For example, Prado et al. (10) conducted a review about the effect of nutrition interventions on muscle status in cancer patients. They summarized that “given the positive findings and theoretical benefits of combining nutrition with other treatments, it is likely that such interventions would be beneficial for individuals with cancer at risk for losing muscle” (10).

In 2014, Grande et al. published a Cochrane Analysis on “exercise for cancer cachexia in adults” with the conclusion that there were no studies to make a qualified statement on effectiveness, acceptability, and safety of multimodal interventions (11). Continued research activity allowed Grande et al. to publish an update of their Cochrane Analysis, including four new trials. But due to bias in most domains, i.e., selection bias or blinding, they were still uncertain to make a statement, referring to another update in the future (12). Further reviews regarding exercise in patients with cancer include those by Allan et al. (13), focusing on exercise and energy regulation in cancer cachexia and Avancini et al. (14), investigating physical activity in patients with lung cancer (13, 14). Both emphasized positive effects of physical activity on, for example, fatigue, QoL, pulmonary function, muscle mass, strength and psychological status. However, Allan et al. pointed out that exercise could increase the gap between energy need and energy intake in patients with cancer cachexia, emphasizing the importance of supporting those patients with nutritional counseling and individual exercise advice (13).

Several reviews about multimodal interventions in advanced cancer patients pointed out that there are positive effects on single components like endurance or depression scores as well as lean mass. The reviews concluded that further high-quality studies are needed in order to give clear recommendations (15, 16).

In recent years, we conducted several combined intervention studies in advanced cancer patients and were not able to achieve the calculated sample size in some of them (17, 18). The reasons for this problem were manifold. For example, many patients could not participate in our trials because they did not meet the inclusion and exclusion criteria. For other candidates, the intervention was too strenuous or not feasible in addition to their cancer disease and treatment. Other researchers made the same experience. A two-arm, open-label, randomized multicenter controlled phase II trial conducted by Pascoe et al. (19) was terminated early due to slow recruitment rates. In this study for patients with advanced lung cancer, all patients received structured nutritional, exercise and symptom control advice. Patients in the intervention group additionally received a nutritional supplement to improve the management of cancer cachexia. The calculated sample size was n = 96 and only n = 38 patients could be recruited in five centers within 1 year. In the intervention group, 9 of 19 patients withdrew from the trial or died of tumor progression (19). In another clinical trial investigating the effect of nutrition and electromyostimulation on gait parameters and physical function in advanced cancer patients, data from only n = 26 patients out of n = 58 in the intervention group could be analyzed. The main reasons for drop-out were a fast deterioration in clinical status, lack of time, death, therapy side effects, surgery or mental stress (20).

For the study at hand we pooled data from two clinical studies to obtain a larger sample size and thus more robust results (17, 18). Using similar methodologies, we had investigated in both trials the effect of a combined therapy including nutritional counseling and physical exercise on nutritional status, QoL, and clinical course in advanced cancer patients.

## 2. Patients and materials and methods

This study used a pooled database of advanced cancer patients prospectively enrolled in two clinical trials. The two trials were designed to investigate the effect of a combined therapy including nutritional counseling and physical exercise on physical performance, nutritional status, body composition, fatigue and QoL. Both studies have been previously published (17, 18). The study protocols were approved by the Cantonal Ethics Committee Zurich (Switzerland) and registered at http://clinicaltrials.gov (NCT01540968 and NCT0285362). Written informed consent was obtained from all patients before study inclusion.

### 2.1. Procedures

Eligibility criteria for the two trials included in this pooled analysis were as follows: patients with metastatic or locally advanced lung or gastrointestinal cancer, an Eastern Cooperative Oncology Group (ECOG) performance status (PS) of ≤ 2 and a life expectancy greater than 6 months as judged by the responsible physician. Patients were considered ineligible if they (i) were on artificial nutrition, (ii) had symptomatic brain metastases or bone metastases or (iii) had had an ileus within the last month.

In the second of the two studies, patients with the following tumor sites were also eligible for inclusion: breast, ovarian, prostate, renal cell, and urothelial. In addition, palliative breast and prostate patients had to be receiving chemotherapy. In the same trial, patients were ineligible if they (iv) had a milk protein allergy and (v) consumed supplements with branched-chain amino acids.

In both trials, the primary investigator enrolled patients and conducted the baseline assessment after written informed consent. After that, patients were randomized using block sizes of six respectively eight. Patients were assigned to either the intervention or the control group at a 1:1 ratio. Patients in the intervention group participated in a nutrition and physical exercise program, while patients in the control group were treated according to the cancer center’s standard medical therapy, following good clinical practice. All parameters were evaluated first at baseline, then 3 months later at the end of the intervention and again 3 months post intervention.

### 2.2. Intervention

#### 2.2.1. Physical exercise

In both trials, the patients in the intervention group conducted two training sessions per week in the hospital’s training facilities. Patients exercised in groups of two to six patients under the supervision of an experienced physiotherapist. One training unit of 90 min included a cardio-pulmonary endurance training either on bicycle-ergometers or on treadmills, and a strength training circuit covering different stations to train all larger muscle groups. The endurance intensity corresponded to a Borg scale-value of four to six (on a scale from zero to ten). When patients were receiving chemotherapy the same day, the intensity was set to a maximum of three on the Borg scale. For the strength part, the training goal was three sets of 10 to 15 repetitions. The strength training workload was adjusted at each session according to the individual patients’ fitness, and participants were instructed to increase resistance as soon as they were able to complete more than 15 repetitions. The second training session at the hospital consisted of a gym training of 60 min with focus on strength, endurance, balance and coordination. The training intensity corresponded to a Borg scale-value of four to five. In the second study, an additional third training session was conducted at home. According to their specific goals, patients could either choose to do an additional strength session with strength bands or an endurance training with walking or cycling for 30 min.

#### 2.2.2. Nutritional counseling

The nutritional intervention by a registered dietitian comprised an extensive initial nutritional assessment followed by individual nutritional measures, i.e., enrichment of foods or energy- and protein-rich snacks. The patients’ nutritional situations were reassessed after 6 weeks and 3 months after the baseline-assessment. Further visits could be arranged as required throughout this period, depending on the clinical and nutritional course. The main objective of the nutritional intervention was for patients to meet protein requirements set at 1.2 g of protein per kg of actual body weight. The energy requirement was calculated according to the Harris-Benedict formula, taking into account factors for disease severity and activity (21). In case of a BMI > 28 kg/m^2^, the energy requirement was calculated using the adjusted body weight. In both trials, nutritional supplements were given to the patients in the intervention group: protein-dense oral nutritional supplements in the first and a leucine-rich whey protein supplement in the second study.

### 2.3. Assessment

#### 2.3.1. Nutritional status

Patients were weighed without shoes and in light clothing. Body composition was assessed using bioelectrical impedance analysis (Body Composition Monitor, Fresenius Medical Care, Switzerland respectively BIA, Akern STA, Florence, Italy). In addition, the nutritional risk screening 2002 (NRS-2002) (22) was conducted. Handgrip strength was measured in the dominant hand using a hydraulic dynamometer (Jamar, Smith and Nephew, Memphis, TN, USA). The test was performed with patients in sitting position holding the elbow flexed at 90° and the forearm and wrist in neutral position. The test was repeated three times with a 1-min rest period between each repetition. The best result of the three measurements was recorded in kilograms (kg) (23, 24).

#### 2.3.2. Dietary intake

After each study assessment, patients were asked to keep a non-consecutive 3-day food diary, including one weekend day, and to record the amount of all ingested foods, beverages, food fortifications, and supplements. The diary was explained with the help of a detailed manual. Volumes and portion sizes were estimated using a photo catalog containing several pictures of serving sizes, which was also handed out to the patients. Portion size was classified into three categories: small, medium, or large. All dietary records were analyzed by the same person, using the software “PRODI 6.2 basis” in the first respectively “6.7 swiss” in the second study (Nutri-Science GmbH, Hausach, Germany).

#### 2.3.3. QoL

Quality of life (QoL) was determined with the European Organization for Research and Treatment of Cancer Quality of Life Questionnaire version 3.0 (EORTC QLQ-C30). The EORTC QLQ-C30 is a 30-item cancer-specific questionnaire including six function scales (physical, emotional, cognitive, social, role and global health status), three symptom scales (fatigue, pain, nausea/vomiting), and six single items assessing the symptoms and financial impact of the disease. Results of the EORTC QLQ-C30 were translated into scores corresponding to a scale of 0 to 100. Higher scores on the function scales indicate better functioning, whereas higher scores on the symptom scales denote impaired functioning (25, 26).

#### 2.3.4. Clinical data

C-reactive protein (CRP), adverse and serious adverse events and unplanned hospital admissions were evaluated based on computerized patient hospital records.

### 2.4. Statistical analysis

Statistical analyses were performed using the programming language R version 3.6.0 (R Foundation, Vienna, Austria). We used Student’s *t*-test to compare changes in values within the 3 and 6 month period, respectively. If variable distribution were not approximately of Gaussian distribution, we applied the Mann-Whitney *U* test. To be able to use all datasets as sensitivity analyses, and since the missing values were assumed not to be completely at random, we used 20-fold multiple imputation by chained equations to estimate the missing values (27) implemented in the package “mice.” The number of imputations was chosen as the maximum percentage of missing variables according to recommendations of White et al. (28). For the imputation, 99 relevant variables were used. *T*-tests for imputed data were done using the packages “MKmisc” and “mitools.” Since numbers within the intervention and control group were small, we also applied regression models to adjust for covariables. Mortality was analyzed using the Cox proportional hazard model with the period between beginning of the trial and death as underlying time-scale.

## 3. Results

In total, 110 patients were included in the pooled analysis (58 from the first and 52 from the second study). The baseline characteristics are shown in Table 1. 68 men (61.8%) and 42 women (38.2%) were randomized either in the intervention (n = 56) or control (n = 54) group. The mean age was 63.0 ± 10.2 years, and the average body mass index (BMI) was 25.3 kg/m^2^. Patients with lung cancer constituted the largest group with (n = 42, 38.2%), followed by patients with colorectal (n = 25, 22.7%) and pancreatic cancer (n = 20, 18.2%). At study inclusion, the groups were well-balanced with regard to demographics, medical characteristics, nutritional status and physical function. Groups were different, though, regarding the days that had passed from first tumor diagnosis to trial start: 419.7 ± 535.5 days for intervention and 772.0 ± 1056.7 days for control patients.

**TABLE 1:** Baseline characteristics of study patients, given are means ± standard deviation, or number with proportion in percent, respectively.

	Total (*n* = 110)	Intervention (*n* = 56)	Control (*n* = 54)
Age (years)	63.0 (± 10.2)	63.0 (± 11.1)	63.0 (± 9.2)
BMI (kg/m^2^)	25.3 (± 4.8)	25.0 (± 4.6)	25.8 (± 4.9)
Days since diagnosis	592.6 (± 847.9)	419.7 (± 535.5)	772.0 (± 1056.7)
**Gender**			
Male	68 (61.8%)	36 (64.3%)	32 (59.3%)
Female	42 (38.2%)	20 (35.7%)	22 (40.7%)
**Site of primary tumor**			
Lung	42 (38.2%)	23 (41.1%)	19 (35.2%)
Colorectal	25 (22.7%)	14 (25.0%)	11 (20.4%)
Pancreatic	20 (18.2%)	9 (16.1%)	11 (20.4%)
Others	23 (20.9%)	10 (17.9%)	13 (24.1%)
**Laboratory parameters**			
CRP (mg/l)	12.0 (± 23.0)	12.8 (± 23.3)	10.9 (± 23.3)
Albumin (g/l)	40.1 (± 4.1)	40.5 (± 4.4)	39.6 (± 3.8)
**Performance status (WHO)**			
0	11 (10.0%)	7 (12.5%)	4 (7.4%)
1	75 (68.2%)	38 (67.9%)	37 (68.5%)
2	21 (19.1%)	11 (19.6%)	10 (18.5%)
Unavailable	3 (2.7%)		

The inter-group difference for changes in BMI, body compartments, NRS, dietary intake, global health status and all symptoms of the EORTC were not statistically significant after 3 and 6 months (Table 2). The inter-group difference for changes in phase angle after 6 months was significant after *t*-test (p = 0.025), but not anymore after adjustment for covariates (Table 2).

**TABLE 2:** Changes of nutritional status and quality of life after 3 and 6 months.

	Baseline		Δ 3 months				Δ 6 months			
	Intervention	Control	Intervention	Control	*p*	95% CI	Intervention	Control	*p*	95% CI
BMI (kg/m^2^)	24.8 ± 4.1	25.9 ± 5.4	0.55	0.28	0.355	−0.83, 0.30	0.64	0.60	0.908	−0.74, 0.66
NRS	2.0 ± 1.0	1.9 ± 1.1	−0.28	0.09	0.092	−0.06, 0.81	−0.34	−0.14	0.241	−0.14, 0.54
Phase angle (°)	5.1 ± 1.0	5.3 ± 0.8	0.06	−0.12	0.199	−0.46, 0.10	**0.12**	**−0.20**	**0.025**	**−0.61, −0.04**
Lean tissue mass (kg)	54.1 ± 21.2	56.2 ± 19.9	−0.10	0.18	0.740	−1.39, 1.95	−0.75	0.26	0.356	−1.16, 3.19
Hand strength (kg)	34.4 ± 10.2	33.9 ± 9.2	**1.79**	**−0.53**	**0.006**	**−3.93, −0.69**	1.25	0.55	0.422	−2.44, 1.03
Energy intake (kcal)	2213.8 ± 689.4	2098.6 ± 738.5	30.14	−142.73	0.251	−470.25, 124.53	−145.41	−229.41	0.560	−370.96, 202.96
Energy intake (%)	97.6 ± 30.9	90.7 ± 33.7	−0.27	−6.89	0.318	−19.71, 6.48	−6.93	−8.32	0.818	−13.41, 10.64
Protein intake (g)	87.8 ± 25.9	79.6 ± 29.9	6.07	−3.65	0.082	−20.69, 1.25	−4.68	−11.24	0.311	−19.43, 6.31
Protein intake (%)	110.7 ± 41.3	95.6 ± 47.2	−10.82	−11.37	0.958	−20.92, 19.83	−23.20	−34.58	0.360	−35.99, 13.24
Carbohydrate intake (g)	241.5 ± 87.9	233.0 ± 91.0	3.56	−0.20	0.845	−41.81, 34.29	−15.12	−12.72	0.895	−33.83, 38.63
Fat intake (g)	90.8 ± 36.5	85.9 ± 34.6	−2.02	−14.45	0.124	−28.33, 3.48	−7.66	−13.24	0.484	−21.45, 10.29
Global health status	65.8 ± 21.0	58.2 ± 19.8	2.16	5.00	0.463	−4.81, 10.50	5.03	4.52	0.913	−9.91, 8.88

NRS, nutritional risk screening; CI, confidence interval; bold = significant.

Importantly, handgrip strength improved significantly from 34.4 ± 10.2 kg at baseline to 36.3 ± 9.9 kg at 3 months in the intervention group compared to 33.9 ± 9.2 kg at baseline to 34.9 ± 9.1 kg at 3 months in the control group (p = 0.006), both after *t*-test as well as after adjustment for covariates (Tables 2, 3).

**TABLE 3:** Regression analysis of change of handgrip strength (kg) after 3 months.

	β	*p*
Handgrip strength baseline	**−0**.**221**	**0.002**
Intervention group	**2**.**702**	**0.002**
Female	**−3**.**672**	**0.005**
Age (years)	**−0**.**101**	**0.025**
CRP (mg/l)	**−0**.**775**	**0.046**

*R*^2^ = 0.190, *p*-value of F-statistic = 0.001, 95% CI = −1.945, 2.107, bold = significant.

Patients in the intervention group joined a mean of 16.3 ± 6.3 of 24 training sessions at the hospital (67.9%). The mean number of individual nutritional counseling sessions was 3.5 ± 1.1 (116.7%). No serious adverse events relating to the nutrition and physical exercise program occurred. There was neither a significant inter-group difference in the average of unplanned hospital admissions nor in the survival time (Table 4).

**TABLE 4:** Analysis of survival time for different tumor diagnoses.

		Parameter estimate	Risk ratio	*p*-value
Lung cancer patients (*n* = 40, events *n* = 33)	Intervention group	0.281	1.325	0.501
	**Female**	**−1**.**000**	**0.368**	**0.016**
	Age (years)	0.005	1.005	0.813
	Days since diagnosis*	**−0**.**506**	**0.603**	**0.002**
	CRP (mg/l)*	**0**.**567**	**1.764**	**0.002**
Colorectal cancer patients (*n* = 21, events *n* = 16)	Intervention group	-0.316	0.729	0.667
	Female	-0.037	0.964	0.959
	Age (years)	0.042	1.043	0.149
	Days since diagnosis*	-0.553	0.593	0.175
	CRP (mg/l)*	0.255	1.290	0.407
Pancreatic cancer patients (*n* = 18, events *n* = 18)	**Intervention group**	**−1**.**191**	**0.304**	**0**.**048**
	Female	1.047	2.848	0.196
	**Age (years)**	**−0**.**089**	**0.915**	**0.027**
	Days since diagnosis*	0.395	1.485	0.303
	CRP (mg/l)*	**0**.**844**	**2.326**	**0.026**

Survival time was analyzed using the Cox propotion hazard model with period between beginning of the trial and death/censoring as underlying time scale, *log-transformed, bold = significant.

The covariates “CRP” and “days from first diagnosis to randomization” were significantly associated with survival time. Patients with higher CRP value had a shorter survival time. A detailed analysis of survival time for the three main diagnoses (lung, colorectal, and pancreatic cancer) showed that in patients with lung cancer, the covariates “CRP value,” “days from first diagnosis to randomization,” and “gender” were significantly associated with survival time. Female patients had a shorter survival time than male patients in our analysis. The analysis for patients with colorectal cancer showed no significant associations at all. Patients with pancreatic cancer randomized to the control group had a 20% shorter survival time than patients in the intervention group (p = 0.048), though.

## 4. Discussion

Data from two randomized intervention trials with advanced cancer patients were included in a pooled analysis regarding nutritional status and survival time. Handgrip strength, as an indicator for muscle strength and associated with short- and long-term mortality and morbidity (24, 29, 30), was the only parameter that showed significant improvement through the implementation of a combined therapy. No significant changes were detected in any of the other parameters, such as BMI, NRS, lean body mass, phase angle, energy, and protein intake, as well as QoL, though. In addition, we observed associations between survival time and several parameters, such as CRP. In our analysis, patients with pancreatic cancer randomized to the intervention group had a 20% longer survival time.

Our results for nutritional status and QoL concur with the results of other trials investigating combined or multimodal therapies in advanced cancer patients (7, 31–33). In line with our results, an improvement in handgrip strength was observed (31) but further effects on muscle mass (7, 32) or QoL could not be detected (34). In contrast to our results, Henke et al. (33) described a clear improvement in physical function (33), and both Schink et al. (34) and Stuecher et al. (35) observed a significantly higher muscle mass (34, 35). Our results emphasize that muscle strength can be affected by a combined therapy including physical exercise due to muscular adaptation, which can lead to a greater increase in muscle strength than in muscle mass (36).

The reasons why multimodal interventions seldom effect significant changes could be multifaceted. Dhillon et al. (32) described the possibility of contamination or selection bias, when patients who were highly motivated to participate in an exercise program started to exercise more, even though they were randomized to the control group. This effect may have a high impact on the results by minimizing inter-group differences (32).

A second reason could be the heterogeneity of our study population. To achieve our sample size, we had to include patients with different diagnoses. Albeit focusing on patients with cancer types that are commonly associated with malnutrition (such as lung or pancreatic cancer), the state of malnutrition or cancer cachexia was no inclusion criteria. Jain et al. (37) investigated “the impact of baseline nutritional and exercise status on toxicity and outcomes in phase I and II oncology clinical trials” and found that patients with baseline malnutrition had poor outcomes. Hence, to strengthen trial results, the baseline nutritional and exercise status should be taken into consideration (37).

A third reason could be a particular imbalance between the study arms in both our trials: for patients in the control group, a substantially longer period had passed between diagnosis and study randomization than for those in the intervention group. Regarding this variable, the randomization inexplicably did not ensure a balanced distribution. On the one hand, it could be speculated that patients who have suffered from their tumor disease for a longer time could be in a worse general condition. On the other hand, these patients could have achieved a more stable general condition. Ultimately, the effect of this imbalance remains unclear.

Fourth, advanced cancer patients are dealing with a dynamic disease situation. Thus, potential positive effects of the intervention on QoL or other aspects might be overridden by the negative impact of disease progression (38).

Fifth, caloric intake and coverage of energy and protein requirements presented a small positive trend for the intervention, but no statistical significance. The large scatter in the data could be one reason for the failed significance. Since the intervention patients showed good adherence to the training and nutritional counselling sessions and adequately implemented the nutritional recommendations, we can rather exclude bad adherence to the study program as a principal reason for the wide scattering of the data. Ester et al. (39) conducted a feasibility trial of a “multimodal exercise, nutrition and palliative care intervention in advanced lung cancer patients.” While they could not find a significant change in energy and protein intake, either, they observed a 75% class attendance, which is in line with our results (39).

In the follow-up analysis after 6 months, no parameter changed significantly between the two groups in comparison to the baseline level. The results of the intervention group seem to converge with the control group, although they have not yet reached the same level. A statement on possible long-term effects cannot be made with our study results.

Even though our nutrition and exercise program showed no significant positive effect on unplanned hospital admissions, adverse events and survival time in our pooled analysis, no negative inter-group impact could be observed, either. This is an important finding with regard to the safety of combined or multimodal programs and in line with several other trials. Combined trials including nutritional and physical therapy seem to be safe and feasible for advanced cancer patients (7, 15, 19, 40).

The patients’ survival time was analyzed depending on the three main diagnoses lung, pancreatic and colorectal cancer in this pooled analysis. We observed a significant association between survival time and the combined intervention in patients with pancreatic cancer. To date, survival has only been analyzed in few studies, and in particular, the impact of a combined program on different tumor diagnoses has not yet been conclusively investigated. Bargetzi et al. (41) conducted “a secondary analysis of a prospective randomized trial, comparing the effect of protocol-guided individualized nutritional support to standard hospital food on the mortality of hospitalized cancer patients.” They found significant improvements in mortality and other outcomes in the intervention group in the short-term. However, interaction tests did not show any significant differences in mortality across the cancer type subgroups (41). In the future, more studies should be conducted with a research focus on survival, as it is undeniably an important outcome.

Three intervention studies are currently ongoing in which multimodal therapy options in cancer patients are investigated: First, the “Multimodal–Exercise, Nutrition and Antiinflammatory medication for Cachexie trial (MENAC)” (7), second, the “Nutrition and Exercise in elderly patients with advanced non-small cell lung or pancreatic cancer study (NEXTAC TWO)” (42) and third, the “Multimodal intervention care on cachexia in patients with advanced cancer (MIRACLE)” (43). We are eagerly awaiting the results of these studies to further discuss our own results, especially because disability free survival is the primary endpoint in the NEXTAC TWO trial (42, 44).

Our pooled analysis has some limitations. First, only two trials could be included, and in both studies, the calculated sample size could not be reached. Notably, the problem of not achieving the sample size and the reasons why patients decline study participation – especially in trials with advanced cancer patients – should get addressed in future studies. Bland et al.’s qualitative study (2022) focused on how people with advanced cancer and cachexia perceive exercise and identified barriers that keep them from exercising, such as, for example, fatigue. They concluded that cancer patients should get offered a combination of home-based and supervised options for exercise: “Combining unsupervised home-based with supervised exercise, which may include incorporating telehealth, may help balance patient exercise preferences that we identified in the current study” (45).

Second, the nutrition and exercise interventions in the two studies were not identical. In the second study, patients were instructed to perform an additional, third exercise session at home, and a leucine-rich supplement was used as part of the nutritional intervention.

The third and main limitation is the imbalance between the two groups. For patients in the control group, a longer period had passed between diagnosis and study randomization than for those in the intervention group, and the influence of this imbalance remains unclear.

In conclusion, the pooled analysis showed a significant improvement in handgrip strength in advanced cancer patients that had participated in a combined therapy. An impaired handgrip strength is an indicator of increased complications during hospital stays and decreased physical status (24). Hence, handgrip strength is associated with mortality and morbidity and is consequently of prognostic significance in hospitalized patients. However, no improvements in further tests were detected. There is great need for further investigations examining the effects of nutritional and exercise therapy, especially on survival time with focus on different cancer diagnoses.

## Data availability statement

The raw data supporting the conclusions of this article will be made available by the authors, without undue reservation.

## Ethics statement

The studies involving human participants were reviewed and approved by Kantonale Ethikkommission Zürich, Stampfenbachstrasse 121, 8090 Zürich. The patients/participants provided their written informed consent to participate in this study.

## Author contributions

PB was the principal investigator in both studies. AU and LS were in charge of the study and data collection. LS and LG were in charge of writing the manuscript. PM conducted statistical analysis. All authors contributed to the analysis of the data, writing of the manuscript, and read and approved the final manuscript.
